# Habitat use and spatial fidelity of male South American sea lions during the nonbreeding period

**DOI:** 10.1002/ece3.2972

**Published:** 2017-04-25

**Authors:** Alastair M. M. Baylis, Rachael A. Orben, Daniel P. Costa, Megan Tierney, Paul Brickle, Iain J. Staniland

**Affiliations:** ^1^ Department of Biological SciencesMacquarie UniversitySydneyNSWAustralia; ^2^ Icelandic Seal CentreHvammstangiIceland; ^3^ South Atlantic Environmental Research InstituteStanleyFalkland Islands; ^4^ Department of Fisheries and WildlifeHatfield Marine Science CenterOregon State UniversityNewportORUSA; ^5^ Department of Ecology & Evolutionary BiologyUniversity of CaliforniaSanta CruzCAUSA; ^6^ School of Biological Science (Zoology)University of AberdeenAberdeenUK; ^7^ British Antarctic Survey NERCCambridgeUK

**Keywords:** foraging site fidelity, juveniles, niche width, *Otaria byronia*, repeatability, resource partitioning, stable isotopes

## Abstract

Conditions experienced during the nonbreeding period have profound long‐term effects on individual fitness and survival. Therefore, knowledge of habitat use during the nonbreeding period can provide insights into processes that regulate populations. At the Falkland Islands, the habitat use of South American sea lions (*Otaria flavescens*) during the nonbreeding period is of particular interest because the population is yet to recover from a catastrophic decline between the mid‐1930s and 1965, and nonbreeding movements are poorly understood. Here, we assessed the habitat use of adult male (*n* = 13) and juvenile male (*n* = 6) South American sea lions at the Falkland Islands using satellite tags and stable isotope analysis of vibrissae. Male South American sea lions behaved like central place foragers. Foraging trips were restricted to the Patagonian Shelf and were typically short in distance and duration (127 ± 66 km and 4.1 ± 2.0 days, respectively). Individual male foraging trips were also typically characterized by a high degree of foraging site fidelity. However, the isotopic niche of adult males was smaller than juvenile males, which suggested that adult males were more consistent in their use of foraging habitats and prey over time. Our findings differ from male South American sea lions in Chile and Argentina, which undertake extended movements during the nonbreeding period. Hence, throughout their breeding range, male South American sea lions have diverse movement patterns during the nonbreeding period that intuitively reflects differences in the predictability or accessibility of preferred prey. Our findings challenge the long‐standing notion that South American sea lions undertake a winter migration away from the Falkland Islands. Therefore, impediments to South American sea lion population recovery likely originate locally and conservation measures at a national level are likely to be effective in addressing the decline and the failure of the population to recover.

## 1 Introduction

The annual cycle of marine vertebrates is organized into breeding and nonbreeding periods. Conditions experienced during the nonbreeding period have profound long‐term effects on individual fitness that can have repercussions at later life‐history stages or carry over to the next breeding period and influence breeding performance and survival (Alves et al., 2013; Bogdanova et al., 2011; Catry, Disa, Phillips, & Granadeiro, 2013; Daunt et al., 2014; Salton, Saraux, Dann, & Chiaradia, 2015). Hence, identifying the distribution of individuals during the nonbreeding period may provide insights into processes that regulate populations and provides information on habitat use, which ultimately enhances the efficiency of conservation and management initiatives (Frederiksen et al., 2011; Ratcliffe et al., 2014).

Pinnipeds are long‐lived marine vertebrates that have a diverse range of life‐history strategies to cope with seasonally variable environments. In otariid seals (fur seals and sea lions), movements during the nonbreeding period range from migrations covering thousands of km (*e.g.,* Northern fur seals *Callorhinus ursinus*), and extended seasonal movements over hundreds to thousands of km (*e.g.,* Antarctic fur seals *Arctocephalus gazella*) to little variation in habitat use over the annual cycle (e.g., Australian sea lions *Neophoca cinerea*; Boyd et al., 1998; Lowther, Harcourt, Page, & Goldsworthy, 2013; Sterling et al., 2014). In addition, female otariids are the sole providers of parental care, meaning that they are typically constrained in foraging trip distance and duration by the fasting ability of their offspring. Accordingly, in species with extended lactation lengths (>6 months), dispersal during the nonbreeding period is often male or juvenile biased, because these sex and age classes are free from central place foraging constraints (Kirkwood et al., 2006; Raum‐Suryan et al., 2004; Robertson et al., 2006; Wright, Tennis, & Brown, 2010).

South American sea lions (SASL) (*Otaria flavescens*) are one otariid species that have an extended lactation length of approximately 10 months (Hamilton, 1934). SASL breed along the Pacific and Atlantic coasts of South America from Uruguay to Peru, and breeding takes place between December and February (Dans et al., 2012; Hamilton, 1934). In recent years, SASL have been the focus of substantial tracking effort, particularly at South Atlantic breeding colonies (Baylis, Orben, Arnould, et al. 2015a,b; Baylis, Orben, et al. 2016a; Hückstädt, Quiñones, Sepúlveda, & Costa, 2014; Riet‐sapriza et al., 2013; Rodríguez et al., 2013; Sepulveda et al., 2015). Nevertheless, considerable knowledge gaps remain. For example, the movement of male SASL during the nonbreeding period (austral autumn, winter and spring) is poorly understood, despite having potentially important implications for conservation and management, including disease transmission and the mediation of genetic exchange between breeding populations, among other factors (Hoffman et al., 2016; Robertson et al., 2006).

Of the few studies that have assessed the nonbreeding movements of male SASL, juvenile male SASL captured in Chile either concentrate their foraging effort in areas associated with salmonoid aquaculture (southern Chile), or in the absence of aquaculture, travel extensively along the Chilean coast (central Chile) (Hückstädt et al., 2014; Sepulveda et al., 2015). Similarly, a bleach marking study revealed that male SASL in Argentina abandon breeding areas during the nonbreeding period and travel extended distances (>500 km) to winter in Patagonia or Uruguay (Giardino et al., 2016). These studies suggest that male SASL show little fidelity to breeding locations and range widely during the nonbreeding period.

At the Falkland Islands, the movements of SASL during the nonbreeding period are of particular interest. SASL at the Falkland Islands experienced a catastrophic population decline between the mid‐1930s and 1965, the cause of which is unclear (Baylis, Orben, Arnould, et al. 2015b). Despite an increase in the number of SASL breeding at the Falkland Islands in recent decades, a population census in 2014 revealed that the number of pups born was <6% of the 1930s estimate and below the number of pups counted in 1965 (Baylis, Orben, Arnould, et al. 2015b). Hence, SASL habitat use during the nonbreeding period may provide insights into impediments to population recovery. However, there is conflicting information on the ecology of SASL at the Falkland Islands during the nonbreeding period.

The IUCN Red List, which is often used to guide national conservation assessments, reports that SASL largely abandon the Falkland Islands during the nonbreeding period, presumably based on anecdotal information from the 1930s (Cárdenas‐Alayza, Crespo, & Oliveira, 2016; Hamilton, 1939). In contrast, recent satellite telemetry data from the Falkland Islands indicate that SASL remain within close proximity to the Falkland Islands (Baylis, Orben, Arnould, et al. 2015b; Baylis, Orben, et al. 2016aa). However, despite recent studies, information on SASL movement and habitat use during the nonbreeding period is rudimentary and incomplete. In the context of a population that has suffered a catastrophic decline, and in light of conflicting information, it is vital to better understand the variability and extent of SASL habitat use during the nonbreeding period.

Here, we focus on males to understand the breadth of SASL habitat use during the nonbreeding period, given that males are free from central place foraging constraints. We assess the habitat use of adult male SASL and juvenile male SASL (broadly defined here as immature males) using the most comprehensive dataset on male SASL to date, that combines both published (Baylis, Orben, Arnould, et al. 2015b; Baylis, Orben, et al. 2016aa) and unpublished data. Specifically, satellite tags were used to characterize movement over the austral autumn and winter. Movement data were integrated with stable isotope analysis of vibrissae to assess habitat use over a longer period of time (years).

## 2 Methods

### 2.1 Animal capture and device deployment

**Figure 1:**
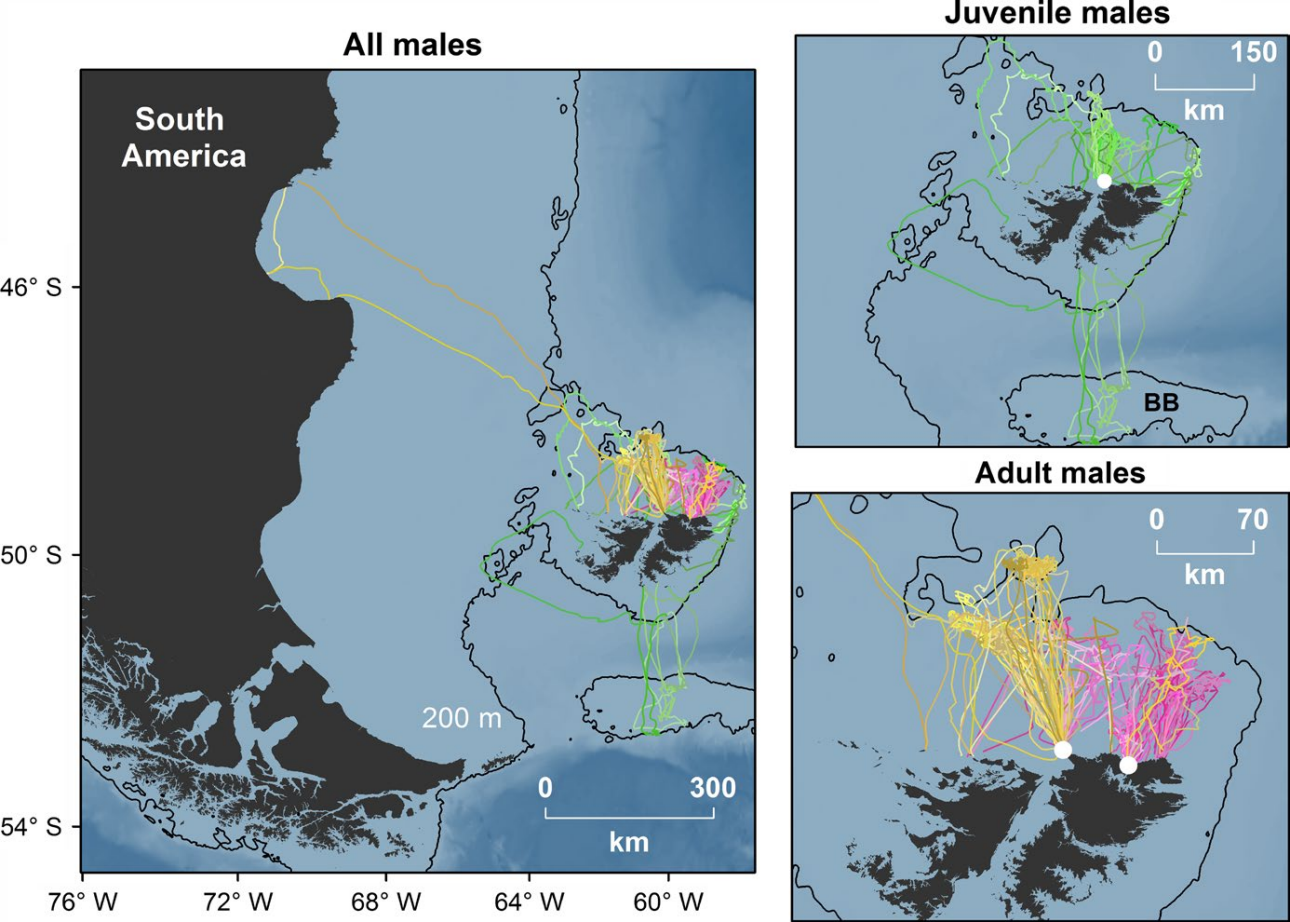
In total, 19 male South American sea lions were successfully tracked from the Falkland Islands between 2011 and 2015 (13 adult males and six juvenile males, see also Supporting information Fig S2). Pink = adult male autumn 2014, Orange = adult male winter 2015, Green = juvenile male winter 2011. Color shades represent different foraging trips. White dots represent the two deployment locations, Cape Dolphin to the west and Big Shag Island to the east. Also presented is the 200 m bathymetric contour. BB = Burwood Bank

SASL were chemically restrained using tiletamine‐zolazepam (Zoletil, Virbac, France), remotely administered using Pneu darts (3.0 cc and 1.0 cc for adult male and juvenile male SASL, respectively) and a CO_2_ powered tranquilizer gun (Dan Inject JM Standard) (Baylis, Page, et al., 2015). Injectable anesthetic drug doses were approx 1.5 mg/kg for adult male SASL, and 3.0 mg/kg for juvenile male SASL. Deployments occurred over three separate years between 2011 and 2015. In May 2011, we deployed Platform Transmitter Terminal (PTT) tags of ARGOS location quality (Sirtrack PTT 101) on six juvenile male SASL at Cape Dolphin (51.24°S, 58.96°W) (Figure 1). A brief summary of these data have previously been published (Baylis, Orben, Arnould, et al. 2015b). Based on size, the age of juvenile male SASL was between 2 and 5 years old. In late February 2014, we deployed PTT tags on 10 adult male SASL at Big Shag Island (52.12°S, 58.92°W; Figure 1). These data have also previously been published, but was a comparison with adult female SASL during pup rearing (Baylis, Orben, et al. 2016a). Finally, in June 2015, we deployed ARGOS linked Fastloc^®^ GPS (Global Positioning System) tags (Wildlife Computer SPOT Tags) on four adult male SASL also at Cape Dolphin. Adult males were distinguished from juvenile males on the basis of body size and the presence of a developed mane. Tags were glued to the back of males using a two‐part epoxy (Devcon 5‐minute^®^ epoxy). Due to logistical constraints, individuals could not be weighed. However, the standard total length and axillary girth of animals were recorded when possible (Table 1).

**Table 1:** Foraging trip characteristics of adult male South American sea lions in autumn (*n* = 9) and winter (*n* = 4), and juvenile male South American sea lions in winter (*n* = 5). Also presented are the mean vibrissae δ^13^C and δ^15^N values

	Adult male autumn (*n* = 9)^a^	Adult male winter (*n* = 4)^b^	Juvenile male winter (*n* = 5)^c^
Total number of foraging trips	39	32	59
Max distance from coast (km)	116	157	346
Min max distance from coast (km)	14	95	39
Mean max distance from coast (km)	74 ± 21	118 ± 18	100 ± 41
Mean total distance traveled (km)	232 ± 74	370 ± 74	282 ± 132
Mean bathymetric depth (m)	120 ± 18	148 ± 22	134 ± 27
Mean foraging trip duration (days)	2.9 ± 0.8	6.9 ± 1.2	4.1 ± 1.4
Max foraging trip duration (days)	4.7	10.8	6.1
Min foraging trip duration (days)	1.8	5.0	2.1
Mean intertrip duration (days)	2.4 ± 0.4	3.2 ± 1.2	2.7 ± 0.7
Mean length (cm)	221 ± 10	207 ± 3	173 ± 14

Stable Isotope values	Male (*n* = 7)	Juvenile (*n* = 6)
	Mean	Mean
Mean δ^13^C (‰)	−13.6 ± 0.2	−13.3 ± 0.8
Mean δ^15^N (‰)	16.8 ± 0.1	17.1 ± 0.9

a One tag failed, leaving location data for nine adult males.

b Metrics exclude the extended movements of one male to Argentina (see Supporting information Table S1).

c One juvenile male stayed within close proximity to land for the duration of deployment. Due to error associated with ARGOS locations, we could not calculate foraging trip metrics (see Supporting information Fig S2).

### 2.2 Location data analysis

The start and end of foraging trips were based on the proximity of locations to land. PTT tags deployed on juvenile males (in winter) and adult males captured during the austral autumn were programmed to transmit every 45 s when at the surface. We first filtered the least squares location processed ARGOS data for erroneous locations using a maximum speed of 3 m/s (Baylis, Orben, et al. 2016a) and the *sdafilter* function in the R package *argosfilter*. The filtered data were then processed using a continuous‐time correlated random walk model that incorporates ARGOS location error for each of the six location classes (3,2,1,0,A,B) within a state‐space model framework (R package *CRAWL*) (Johnson, London, Lea, & Durban, 2008).

GPS tags were programmed to acquire locations every 10 minutes. Initial GPS data exploration indicated that locations estimated by only four satellite acquisitions were unreliable, and these were removed prior to analysis. Data exploration also revealed that the outbound and inbound sections of foraging trips typically had large temporal and spatial gaps between GPS locations. To avoid the assumption of linear movement between GPS locations, we also processed our GPS data using the *CRAWL* package. Hence, while the GPS locations were retained in the model and assumed to represent the true location of the animal, we still generated 1000 simulated tracks, which allowed for error in movement to be integrated between GPS locations. We interpolated hourly locations for both GPS and PTT data using the “best‐fit” track produced by the model. Hourly locations were then used to calculate foraging trip metrics.

For each foraging trip, we calculated foraging trip duration, intertrip duration (time between consecutive foraging trips), maximum distance from the Falkland Islands, and mean bathymetry (GEBCO_14 30 arc second dataset, extracted for each hourly location along a foraging track using ArcMap) (ArcGIS 10.4, Redlands, CA, USA). Bathymetry was not included in the analysis because it was correlated with distance. To test whether distance and duration varied over time, we used linear mixed effects models (LME) with a restricted maximum‐likelihood (REML) implemented using the R package *nlme*. Individual was included as a random effect, and Julian day of as the covariate. Julian day was first standardized by subtracting the mean for each value and dividing by the SD. Model validation was performed by plotting Pearson's residuals and fitted values (Zuur, et al. 2009). We also tested whether differences existed in distance and duration between groups (adult male autumn, adult male winter, and juvenile male) using LME, with group as a factor. Post hoc multiple comparison tests were performed using Tukey's pairwise comparisons (R package *multicomp*). Our study design did not permit season, site, age, or year effects to be separated.

### 2.3 Repeatability and individual foraging site fidelity

To characterize consistency in foraging trips, we used simple measures of maximum foraging trip distance and foraging trip duration. To explore within versus between individual variance for trip distance and duration, we used LME with REML implemented using the R package *rptR* (Nakagawa & Schielzeth, 2010). The repeatability measure *R* is a ratio of variances that ranges from 0 (low repeatability, high within‐individual variance) to 1 (high‐repeatability, low within‐individual variance). We considered *R* values >.50 as highly repeatable (Potier et al., 2015). Uncertainty was quantified via parametric bootstrapping (*n* = 1000 times) and repeatability was assessed at the individual level. Our model included group as a fixed effect (comprising adult male autumn, adult male winter, and juvenile male) and individual as a random effect. Model residuals were inspected to ensure model assumptions were met. We also calculated the number of haul out sites used and the proportion of foraging trips that started and ended at the same site.

To quantify individual foraging site fidelity, we first estimated kernel utilization distributions for each foraging trip using the R package *adehabitat* (Calenge, 2006). Smoothing parameters (h) for the kernel analyses were calculated using the ad hoc method (Worton, 1989). We then calculated the average overlap between all combinations of an individual's foraging trips, and overlap between successive foraging trips using Bhattacharyya's affinity (BA) (Fieberg & Kochanny, 2005; Wakefield et al., 2015). Our earlier work highlights that SASL foraging trips are typically restricted to the Patagonian Shelf (Baylis, Orben, Arnould, et al. 2015a; Baylis, Orben, et al. 2016a). This implies that foraging site fidelity may arise from chance alone (i.e., restricted foraging trip distance increases the probability of overlap), rather than an individual level trait per se. To test the null hypothesis that overlap between an individual's foraging trips was not greater than expected by chance, we ran a randomization analysis (Breed, Bowen, & Leonard, 2013). Using a dataset that contained overlap between all possible combinations of foraging trips (irrespective of individual), we randomly sampled overlap in foraging trips to create dataset of the same length for comparison. This was then permuted 1,000 times without replacement. Significance was determined by the proportion of random overlaps that were smaller than the observed overlap, so that if the observed overlap was larger than all 1,000 randomly generated overlaps, then *p* ≤ .001 (Breed et al., 2013).

### 2.4 Stable isotope analysis

Stable isotopes ratios are commonly used to infer the trophic niche of marine predators, with carbon values providing a proxy of foraging habitat and nitrogen values a proxy of trophic level (Newsome, Clementz, & Koch, 2010). Stable isotope ratios of metabolically inert tissues, such as vibrissae, remain unchanged once grown. Hence, longitudinal sampling of vibrissae provides information on an individual's trophic and spatial history (McHuron et al., 2015; Newsome et al., 2010). Vibrissae were collected from SASL by cutting the largest one as close to the skin as possible. Vibrissae length ranged from 123 to 264 mm for adult male SASL and 75 to 155 mm for juvenile male SASL. Male otariid vibrissae grow linearly over time, but available data indicate that the average growth rate of vibrissae varies between and within species (McHuron et al., 2015; Rea et al., 2015). No vibrissae growth estimates are available for male SASL, but given mean growth rates for other sea lion species range 0.02–0.29 mm/day, we presume that our study integrates diet over a period of years (McHuron et al., 2015; Rea et al., 2015).

We analyzed all juvenile male (*n* = 6) and a random subset of adult male vibrissae (*n* = 7, sample size was limited by available funding). Stable isotope values of adult male SASL vibrissae have previously been published (Baylis, Orben, et al., 2016a). Prior to analysis, SASL vibrissae were cleaned using a sponge and placed in an ultrasonic bath of distilled water for 5 minutes. Vibrissae were dried using 95% ethanol and inspected under a microscope to ensure they were clean. If necessary, the cleaning process was repeated. Vibrissae were then cut into 5–mm‐long consecutive segments starting from the proximal (facial) end. To produce a meaningful isotopic measurement, our target mass for each vibrissae segment was 0.5 mg. To achieve our target mass, it was necessary to subsample each 5‐mm section. Samples were packed in tin containers, and carbon and nitrogen isotope ratios were determined by a Carlo‐Erba elemental analyzer interfaced with a Finnigan Delta Plus XP mass spectrometer (Stable Isotope Laboratory, University of California Santa Cruz, USA). Stable isotope ratios were measured in parts per mille ‰ deviation from international standards (V‐PDB for carbon and AIR N2 for nitrogen), according to the following equation δ X  =  [(Rsample/Rstandard) – 1] × 1000 where X is 15N or 13C and R is the corresponding ratio of (15N/14N) or (13C/12C). Stable isotope ratios are reported as δ13C values for carbon and δ15N values for nitrogen. Data were corrected for sample mass and instrument drift. Measurement precision (standard deviation), based on within‐run replicate measures of the laboratory standard (pugel), was 0.03 ‰ for δ^13^C and 0.06 ‰ for δ^15^N isotope values.

Initial data exploration revealed a large drop in δ^15^N values (~2 ‰) along three juvenile male SASL vibrissae that was indicative of a weaning signal (Figure S1). Hence, some juvenile male SASL vibrissae integrated both preweaning (*i.e.,* their mothers diet) and postweaning dietary information. To objectively determine the location along the whisker where δ^15^N mean values changed, we performed a change point analysis using the R package *changepoint* (Killick & Eckley, 2014). We present both the complete vibrissae stable isotope values and the subset of vibrissae stable isotope values, which we assumed reflected nutritional independence.

We compared adult and juvenile male SASL δ^13^C and δ^15^N isotope values using a LME, with individual included as a random effect and a low order correlation structure (corARMA, *p* = 2) to account for temporal autocorrelation. To examine differences in the isotopic niche width of adult male and juvenile male SASL, we used mean stable isotope values for each individual to calculate standard ellipse areas (SEA) and convex hulls. SEA are a proxy for core isotopic area (analogous to SD for univariate data and contain approximately 40% of the data), while convex hulls represent the total niche area occupied (Jackson, Inger, Parnell, & Bearhop, 2011). SEA were calculated within a Bayesian framework, and uncertainty in ellipse area (credible intervals) calculated using 100 000 posterior draws (Jackson et al., 2011). We also calculated the mean distance to centroid (CD) and standard deviation of nearest‐neighbor distance (SDNND), as a measure of the diversity of individual niches and evenness of spacing within a niche space, respectively (Layman, Arrington, Montana, & Post, 2007). Low CD values suggest lower diversity, while low SDNND values suggest a more even distribution of trophic niches (Layman et al., 2007). In addition, to assess individual variability, we used the two‐dimensional (δ^13^C and δ^15^N) isotopic space occupied by each individual as a proxy for variability in diet and habitat use (Baylis, Orben, et al., 2016a; Elliott Smith, Newsome, Estes, & Tinker, 2015). Specifically, we calculated SEA (as described above) for each individual SASL, based on sequential vibrissae segments. All stable isotope metrics were calculated using the R Package *SIBER* (Jackson et al., 2011).

## 3 Results

We deployed 20 satellite tags on male SASL, including 14 tags on adult male SASL and six tags on juvenile male SASL. One adult male tag failed, leaving location data for 13 adult male SASL. Adult male SASL length was significantly longer than juvenile males (Table 1; Welch's *t* test *t* = 7.3, *df* = 9.2, *p* < .001). In total, 130 complete foraging trips were recorded from late February to late October (Supporting information Table S1). This included 71 foraging trips from adult male SASL (*n* = 39 in autumn and *n* = 32 winter) and 59 foraging trips from juvenile male SASL (Table S1). Deployment duration for adult males ranged from 9 to 33 days for autumn and 13 to 136 days for winter. Deployment duration for juveniles over winter ranged from 36 to 153 days (Table S1).

Male SASL foraging trips were restricted to the Patagonian Shelf (including the Burwood Bank) and Patagonian Shelf slope (Figure 1). One juvenile male SASL remained within close proximity to land for the duration of the deployment period (max distance from land 13.5 km, mean 1.2 ± 1.8 km). We could not reliably define the start and end of foraging trips for this individual. Therefore, it was excluded from movement analysis (see Figure S2). One adult male (148756) undertook an 800 km trip to the San Jorge Gulf, Argentina and returned to the Falkland Islands 6.5 weeks later (Figure 1, Table S1). This extended trip was an outlier and excluded from the analysis of foraging trip metrics. However, its implications are discussed.

On average foraging trips were short in distance and duration (127 ± 66 km and 4.1 ± 2.0 days, respectively) and did not increase significantly over the deployment period (LME: *p* > .05 for all comparisons). Overall, maximum foraging trip distance was not significantly different between groups (LME: *F*_2,15_ = 3.0, *p* = .079), although on average adult males in autumn travelled over 1.5 times further than adult males in winter (Table 1). Foraging trip duration did, however, differ (LME: *F*_2,15_ = 17.9, *p* < .001), with pairwise comparisons revealing that adult males in winter had significantly longer foraging trip durations than both adult males in autumn and juvenile males (Table 1; *Z*_adult autumn‐winter_ = 5.9, *p* < .001 and *Z*_adult autumn‐juvenile_ = −3.9, *p* < .001). Intertrip duration was not significantly different between groups (LME: *F*_2,15_ = 2.6, *p* = .107). Finally, adult male and juvenile male SASL tracked over winter (captured at Cape Dolphin, but in different years) foraged in similar areas (95% BA = 0.65), but had low overlap in core foraging areas used (50% BA = 0.11).

### 3.1 Repeatability and individual foraging site fidelity

Adult males started and ended at the same location on 58 ± 41% of foraging trips (autumn mean 60 ± 38% and winter mean 54 ± 54%). While juvenile males started and ended foraging trips at the same location on 76 ± 22% of foraging trips. Male SASL typically hauled out at a limited number of locations (mean adult males: 2 ± 1 haul out locations, range 1–3 (excluding the return foraging trip to Argentina) and mean for juvenile males: 3 ± 4 haul out locations, range 1–12). Overall, repeatability in foraging trip distance and duration was high for males (duration: *R *=* *.51, 95% CI = [0.25, 0.68], *p* < .01 and distance: *R *=* *.68, 95% CI = [0.43, 0.81], *p* < .01). Individual males also typically showed a high degree of foraging site fidelity (based on consecutive foraging trips, all foraging trip combinations, or both), which was greater than expected by chance alone (Table 2). The lowest BA values were for juvenile male 6074 that circumnavigated the Falkland Islands and foraged as far south as the Burwood Bank (Figure 1).

**Table 2:** For each individual South American sea lion, we calculated 95% utilization distributions for each foraging trip. We used Bhattacharyya's affinity (BA) to assess overlap between consecutive foraging trips, and overlap between all combinations of foraging trips. We ran a randomization analysis to test the null hypothesis that overlap between individual foraging trips was not greater than expected by chance alone. *p‐*Values were determined by the proportion of random overlaps that were smaller than the observed overlap

Male id	Foraging trips	Mean BA (consecutive foraging trips)	*p*‐value	Mean BA (all foraging trip combinations)	*p*‐value
Juvenile					
1543	7	0.59 ± 0.16	<.001	0.55 ± 0.13	<.001
2162	19	0.63 ± 0.18	<.001	0.62 ± 0.19	<.001
6074	19	0.23 ± 0.27	>.05	0.11 ± 0.20	>.05
68025	9	0.57 ± 0.22	<.001	0.31 ± 0.23	>.05
103751	5	0.55 ± 0.28	<.001	0.39 ± 0.24	>.05
Adult autumn					
112937	4	0.31 ± 0.25	>.05	0.31 ± 0.29	>.05
112938	3	0.55 ± 0.27	.004	0.47 ± 0.27	.038
112939	6	0.33 ± 0.27	>.05	0.35 ± 0.25	>.05
112940	3	0.60 ± 0.23	.003	0.52 ± 0.20	.010
112941	4	0.45 ± 0.29	<.001	0.48 ± 0.27	<.001
112942	10	0.56 ± 0.24	<.001	0.49 ± 0.25	<.001
112943	4	0.79 ± 0.12	<.001	0.74 ± 0.10	<.001
112944	2	0.55	>.05	0.75 ± 0.29	.010
112945	3	0.37 ± 0.13	>.05	0.44 ± 0.15	>.05
Adult winter					
148754	2	0.95	<.001	0.85 ± 0.14	<.001
148755	15	0.73 ± 0.13	<.001	0.70 ± 0.15	<.001
148756	7	0.30 ± 0.36	>.05	0.25 ± 0.29	>.05
148759	8	0.78 ± 0.15	<.001	0.74 ± 0.12	<.001

### 3.2 Stable isotope analysis

**Figure 2:**
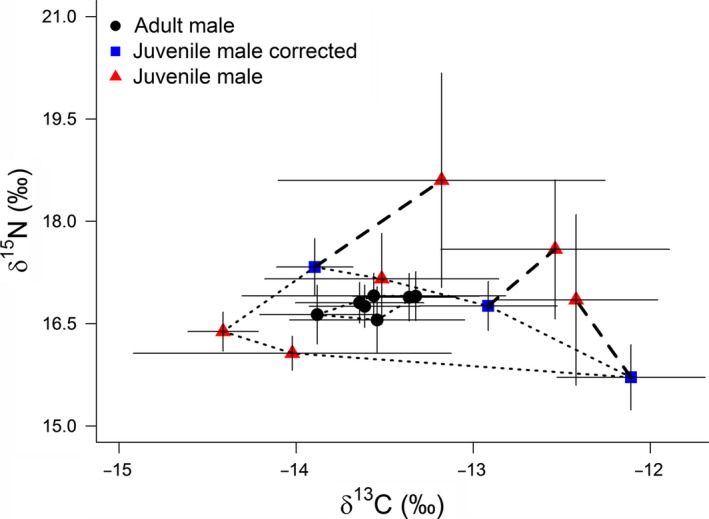
Stable Isotope values of 13 South American sea lion vibrissae (*n* = 7 adult male and *n* = 6 juvenile male). Juvenile males had a larger range of δ^13^C and δ^15^N values. Three juvenile male SASL vibrissae integrated diet over both the pre‐ and postweaning period. Hence, for three males, we also present corrected stable isotope values that reflect the period postweaning. Bi‐plots represent individual means ± SD, while convex hulls (thin dashed line) represent total niche area for adult males and juveniles, respectively

We analyzed 13 whiskers (*n* = 7 adult male SASL captured during the austral autumn and *n* = 6 juvenile male SASL) representing 355 whisker segments (*n* = 236 for adult male SASL and *n* = 119 for juvenile male SASL). Overall, mean δ^13^C and δ^15^N values were not significantly different between adult male and juvenile male SASL—irrespective of whether all isotope values were analyzed (LME δ^13^C values: *df* = 11, *t* = 1.68, *p* = .12 and LME δ^15^N values: *df* = 11, *t* = 1.15 *p* = .26), or just the subset of δ^13^C and δ^15^N values associated with the postweaning period (LME δ^13^C values: *df* = 11, *t* = 1.68, *p* = .12 and LME δ^15^N values: *df* = 11, *t* = 1.15 *p* = .26, respectively) (Table 1; Figure 2). Nevertheless, overall the isotopic niche area of adult male SASL was smaller than juveniles (SEA was twice as small—probability 94% based on posterior SEA, and the total isotopic area was also 94% smaller) (Table 3). Similarly, individual adult male SASL had significantly smaller SEA than individual juvenile male SASL (Welch's *t* test: *t* = 3.0 *df* = 6.3, *p* = .023) (Table 3; Figure S3). Lower SDNND and CD values for adult male SASL indicated that mean individual male isotope values were more consistent and more evenly distributed, when compared to juvenile male SASL (Table 3, Figure 2).

**Table 3:** Stable isotope δ^13^C and δ^15^N values of individual South American sea lion vibrissae were used to calculate standard ellipse area (SEA), total area (TA), centroid distance (CD), and standard deviation nearest‐neighbor distance (SDNND) (see Section 2 for details)

	Adult male	Juvenile male
Individual SEA (‰)	0.64 ± 0.16	1.18 ± 0.41
Group SEA (‰)	1.08	2.50
Group SEA 95% CI (‰)	0.43–1.92	95% CI: 0.9–4.64
TA (‰)	0.10	1.80
SDNND	0.10	0.35
CD	0.19	0.53

SEA were calculated for each age class (adult and juvenile) and for each individual. These metrics revealed that juvenile male South American sea lions had a larger isotopic niche when compared to adult males. 95% CI = 95% Credible Intervals.

## 4 Discussion

Male SASL at the Falkland Islands typically used several haul out sites during the nonbreeding period. Nevertheless, our data clearly showed that, over three separate years, individual males typically behaved like central place foragers and had a high degree of repeatability in foraging trip metrics (distance and duration) and to a lesser degree, individual fidelity to foraging sites. This is despite not being constrained by the need to care for dependent young ashore. Although SASL males at other breeding sites (Giardino et al., 2016), and males of other sea lion species (Raum‐Suryan et al., 2004; Robertson et al., 2006), undertake extended movements during the nonbreeding period, only one male SASL in our study undertook an extended foraging trip away from the Falkland Islands. All other foraging trips where short in distance and duration (127 ± 66 km and 4.1 ± 2.0 days, respectively) and cannot be characterized as extended seasonal movements. This suggests that male SASL at the Falkland Islands do not undergo a seasonal range expansion in foraging habitat. Accordingly, our results do not support the long‐standing assumption that the majority of SASL abandon the Falkland Islands over winter—at least with regard to males (Cárdenas‐Alayza et al., 2016; Hamilton, 1939). Males and young animals are often regarded as the main drivers of breeding range expansion and gene flow (Greenwood, 1980; Raum‐Suryan et al., 2004; Robertson et al., 2006). While the ecological implications of restricted male movements could be profound and include limited male‐mediated gene flow, SASL dispersal capability clearly exists. Locally, at the Falkland Islands, male SASL could move between breeding colonies during the breeding season, given many of the over 70 breeding locations are within close proximity to one another (Baylis, Orben, Arnould, et al. 2015b).

Males of several other nonmigratory otariid species also show unexpected central place foraging tendencies that are broadly consistent with the behavior of male SASL that we describe, challenging the assumption that male life‐history promotes dispersal (Kernaléguen et al., 2015; Lowther et al., 2013; Page et al., 2006; Staniland & Robinson, 2008). Hypotheses proposed to explain the central place foraging behavior of male otariids include energy maximization strategies, predator avoidance, molt, or that it enables males to gain knowledge about breeding areas and establish a hierarchy (Baylis, Orben, et al. 2016a; Kernaléguen et al., 2015; Kirkwood et al., 2006; Page, McKenzie, & Goldsworthy, 2005; Staniland & Robinson, 2008). Molt can be excluded because the period over which male SASL were tracked was either prior (autumn) or postmolt (winter). Of the remaining hypothesis, territory holding/breeding hierarchy is the least parsimonious. Although higher reproductive success in some species is achieved by being familiar with neighbors (i.e., the fitness impacts of the dear enemy effect) (Beletsky & Orians, 1989) and SASL males in autumn attended breeding colonies, SASL males in winter gained no insight into breeding areas or “dear enemies,” because breeding beaches at Cape Dolphin are abandoned over winter (Hamilton, 1939; A. M. M. Baylis pers. obs.). Furthermore, male SASL, like all male otariids, are capital breeders and establish a hierarchy in the weeks prior to the start of the breeding season (Hamilton, 1934). This is a period when male aggression peaks, and body mass and body condition are greatest (Pérez‐Alvarez, Carrasco, Sepúlveda, & Quiñones, 2013). Presumably, any hierarchy established during the nonbreeding season, when SASL males are not aggressive and haul out alone or in small groups of mixed ages, has little consequence on the outcome of the breeding season. Similarly, the restricted movements of adult male Australian sea lions (*Neophoca cinerea*) are thought to be a consequence of their asynchronous breeding cycle (Lowther et al., 2013). This too seems unlikely, given that adult male SASL also had restricted movements, yet SASL have a synchronous breeding cycle (Hamilton, 1934).

Rather, given that successful foraging underpins animal survival and that short‐term decisions can influence long‐term fitness, it is intuitive that the foraging behavior of a capital breeder is shaped by processes that influence foraging success and minimize the chance of starvation (McNamara & Houston, 1996; Stephens & Krebs, 1986). Hence, a more parsimonious hypothesis to explain the central place foraging behavior and consistency in habitat use that we report is that SASL used foraging strategies that maximized net energy gain and minimized predation risk (Stephens, 1981). Given predation risk for SASL at the Falkland Islands is presumably low (Baylis, Orben, Arnould, et al. 2015b), key factors that determine SASL foraging behavior are likely to be related to resource availability and predictability, and thermoregulatory costs.

In particular, the foraging site fidelity that we report is expected to have profound consequences for individual fitness (Piper, 2011). It is often presumed that long‐lived animals benefit from familiarity with resources, because familiarity facilitates direct travel to foraging areas (reducing the energetic costs of travel) and confers energetic advantages over an individual's lifetime (Baylis, Page, McKenzie, & Goldsworthy, 2012; Baylis, Orben, Pistorius, et al., 2015; Bradshaw, Hindell, Sumner, & Michael, 2004; Merkle, Cherry, & Fortin, 2015; Robson et al., 2004; Wakefield et al., 2015). Given male SASL foraging trips were restricted to the Patagonian Shelf, presumably reflecting a preference for benthic foraging, and that the Patagonian Shelf Large Marine Ecosystem is one of the most productive regions in the world's oceans (Acha et al., 2004), a high degree of foraging site fidelity is not surprising. Foraging site fidelity is likely to be an optimal foraging strategy for SASL if prey resources are associated with spatially predictable ocean features, such as the Patagonian Shelf slope (*i.e*., bathymetry), and associated frontal zones (Acha et al., 2004; Arkhipkin, Brickle, & Laptikhovsky, 2013). Indeed, static ocean features such as the Patagonian Shelf slope may help to explain the similarity in male SASL habitat use in different years.

A potential cost of foraging site fidelity is that individuals lack the flexibility to respond to environmental change (Bolnick et al., 2003; Wakefield et al., 2015). However, we did not detect foraging site fidelity in all males tracked, and the emerging picture from other male SASL satellite telemetry and marking studies is that male SASL nonbreeding movements are diverse and complex (ranging from foraging site fidelity to extended seasonal movements) (Giardino et al., 2016; Hückstädt et al., 2014). This implies that male SASL have a high degree of behavioral flexibility. Intuitively, behavioral differences between breeding sites (*i.e.,* Falkland Islands, Argentina, Chile) reflect differences in the predictability or availability of preferred prey. For example, seasonal changes in the use of haul out sites by Steller sea lions (*Eumetopias jubatus*) are linked to the availability of prey near haul out sites (Womble, Sigler, & Willson, 2009). In addition, differences in male SASL behavior between breeding sites (site fidelity verses seasonal movements) could also represent distinct phenotypic variations. For example, environmental conditions experienced during early growth and development could shape and constrain male SASL foraging behavior, given individual experiences during animal development are important in phenotypic development (Bateson, 2015; Monaghan, 2008). Interannual tracking combined with environmental monitoring is ultimately required to better understand the proximate causes of male SASL nonbreeding behavior. Given the potential implications of nonbreeding movements on individual fitness and eco‐evolutionary dynamics, future studies should ideally be regional in scope and simultaneously deploy biologging tags at several SASL breeding sites.

Our study was the most comprehensive male SASL satellite telemetry study to date. However, we lacked the power to differentiate between the confounding effects of age, site, year, and season. For example, although the mean maximum distance travelled by adult male SASL during winter was on average further when compared to autumn, this may have been an artifact of deployment site. Specifically, the distance to the Patagonian Shelf slope from Cape Dolphin (winter deployment location) is further when compared to Big Shag Island (autumn deployment location). Nevertheless, although our sampling design did not permit behavioral differences to be investigated in detail, foraging strategies are often dependant on animal age and size (Stephens & Krebs, 1986). Therefore, we might expect clear differences in the foraging behavior of adult and juvenile male SASL, and for these differences to be amplified during the nonbreeding period, as is reported for other marine predators (Breed, Bowen, & Leonard, 2011; Daunt et al., 2007; Page et al., 2006).

For example, despite their higher absolute energetic requirements, adult male SASL should be more efficient foragers than juvenile male SASL because age and body size are often correlated with enhanced diving capacity and performance (Weise, Harvey, & Costa, 2010). As resources around a central place become limited, the different energetic costs and foraging efficiencies of adult and juvenile male SASL should be reflected either in habitat choice, or in foraging trip distance and duration. We found no significant differences in foraging trip distance and intertrip duration. However, adult male SASL had longer foraging trip durations than juvenile male SASL over winter. This result was surprising because, in other pinniped species, adult males typically have shorter foraging trips than juvenile males and this is thought to reflect higher foraging efficiency and the competitive advantage associated with a larger body size (Breed et al., 2011; Page et al., 2006; Weise et al., 2010). Differences in foraging trip durations between adult male and juvenile male SASL over winter could simply reflect interannual differences in resource availability, given deployments occurred across different years (2011 and 2015). An alternative explanation is that adult male SASL are more selective when searching for prey, while juvenile males are more opportunistic, and utilize a broader range of prey species. Unfortunately, data on male SASL prey do not exist for the Falkland Islands, and we did not collect dive data, both of which would have improved our understanding of adult and juvenile male foraging behavior and foraging efficiency. However, the results from our stable isotope analysis do provide some insights into dietary specialization.

The isotopic niche of adult male SASL and juvenile male SASL overlapped. However, adult male SASL had a significantly smaller isotopic niche than juvenile male SASL. This suggests that individual adult male SASL consistently foraged in similar habitats and at similar trophic levels. This interpretation assumes that the prey groups consumed by adult male SASL had distinct stable isotope values, meaning that dietary variation was not underestimated. In contrast to adult male SASL, juvenile male SASL had a significantly larger niche (even after accounting for a weaning signal), implying a broader range of individual foraging strategies. For example, the stable isotope values of juvenile male SASL indicated that they foraged inshore, offshore, or alternated between these two habitats, as has previously been reported for adult female SASL (Baylis, Orben, Arnould, et al. 2015a; Baylis, Kowalski, Voigt, et al., 2016a). It is not unusual for adult diet to be less diverse when compared to juveniles, and this may reflect adults acquiring more specialized foraging strategies with age and experience, when compared to the more generalist strategies in younger, less experienced individuals (Beck, Bowen, McMillan, & Iverson, 2003; Polis, 1984). Hence, the larger isotopic niche of juvenile male SASL may reflect juvenile males utilizing a wide range of foraging strategies to compensate for a lack of proficiency or lack of experience. In addition, if intraspecific competition between males reduced the availability of preferred prey resources, then the niche variation hypothesis could help to explain the more diverse isotopic niche of juvenile male SASL, which presumably are outcompeted by larger adult males (Bolnick et al., 2003).

Finally, given that SASL typically remained at the Falkland Islands during the nonbreeding period, impediments to population recovery (either anthropogenic or via natural processes) are likely to be manifested or originate locally, rather than elsewhere. Hence, conservation initiatives at the Falkland Islands are vital to addressing the population decline and the failure of the population to recover. Fisheries and environmental change are prevailing factors that influence pinniped population dynamics globally (Kovacs et al., 2012). Therefore, future research that aims to assess impediments to population recovery should quantify trophic and operational interactions between SASL and commercial fisheries and assess the influence of interannual environmental variability on SASL habitat use.

## Conflict of Interest

None declared.
