# Supplementary material for: Habitat use and spatial fidelity of male South American sea lions during the nonbreeding period
Source: Ecol Evol. 2017 Apr 25;7(11):3992–4002. doi: 10.1002/ece3.2972 (PMC5468127; doi:10.1002/ece3.2972)
Supplement: Supplementary file 1 [file ECE3-7-3992-s001.docx]

**Table S1:** Biologging tags were successfully deployed on 18 male South American sea lions (*Otaria flavescens*) breeding at the Falkland Islands. ID 6074 circumnavigated the Falkland Islands and ID 148756 undertook an extended foraging trip to Argentina.

| \| **Year** \| **Age** \| **ID** \| **Trip** \| **Trip start** \| **Trip end** \| **Total distance (km)** \| **Max distance (km)** \|  \| **Inter-trip duration (days)** \| \| --- \| --- \| --- \| --- \| --- \| --- \| --- \| --- \| --- \| --- \| \| **Trip duration (days)** \| \| 2014 \| Adult \| 112937 \| 1 \| 23/02/2014 14:48 \| 26/02/2014 1:59 \| 205 \| 95 \| 2.5 \|  \| \| 2014 \| Adult \| 112937 \| 2 \| 2/03/2014 6:44 \| 4/03/2014 6:40 \| 182 \| 81 \| 2 \| 4.2 \| \| 2014 \| Adult \| 112937 \| 3 \| 6/03/2014 9:10 \| 8/03/2014 7:18 \| 131 \| 63 \| 1.9 \| 2.1 \| \| 2014 \| Adult \| 112937 \| 4 \| 11/03/2014 0:00 \| 13/03/2014 20:18 \| 256 \| 103 \| 2.8 \| 2.7 \| \| 2014 \| Adult \| 112938 \| 5 \| 28/02/2014 5:36 \| 3/03/2014 8:07 \| 238 \| 90 \| 3.1 \|  \| \| 2014 \| Adult \| 112938 \| 6 \| 5/03/2014 0:12 \| 8/03/2014 5:38 \| 239 \| 60 \| 3.2 \| 1.7 \| \| 2014 \| Adult \| 112938 \| 7 \| 11/03/2014 0:01 \| 13/03/2014 4:05 \| 211 \| 64 \| 2.2 \| 2.8 \| \| 2014 \| Adult \| 112939 \| 8 \| 21/02/2014 7:53 \| 24/02/2014 9:38 \| 231 \| 75 \| 3 \|  \| \| 2014 \| Adult \| 112939 \| 9 \| 28/02/2014 8:16 \| 28/02/2014 13:36 \| 24 \| 14 \| 0.2 \| 3.9 \| \| 2014 \| Adult \| 112939 \| 10 \| 2/03/2014 5:11 \| 5/03/2014 1:09 \| 237 \| 74 \| 2.8 \| 1.6 \| \| 2014 \| Adult \| 112939 \| 11 \| 7/03/2014 8:47 \| 9/03/2014 5:20 \| 150 \| 53 \| 1.8 \| 2.3 \| \| 2014 \| Adult \| 112939 \| 12 \| 10/03/2014 8:26 \| 14/03/2014 0:27 \| 280 \| 83 \| 3.7 \| 1.1 \| \| 2014 \| Adult \| 112939 \| 13 \| 16/03/2014 12:09 \| 21/03/2014 4:41 \| 272 \| 57 \| 4.7 \| 2.5 \| \| 2014 \| Adult \| 112940 \| 14 \| 4/03/2014 6:15 \| 8/03/2014 16:11 \| 378 \| 116 \| 4.4 \|  \| \| 2014 \| Adult \| 112940 \| 15 \| 11/03/2014 4:47 \| 15/03/2014 5:55 \| 324 \| 105 \| 4 \| 2.5 \| \| 2014 \| Adult \| 112940 \| 16 \| 18/03/2014 8:42 \| 23/03/2014 0:42 \| 341 \| 93 \| 4.7 \| 3.1 \| \| 2014 \| Adult \| 112941 \| 17 \| 24/02/2014 9:49 \| 28/02/2014 5:16 \| 267 \| 93 \| 3.8 \|  \| \| 2014 \| Adult \| 112941 \| 18 \| 1/03/2014 9:42 \| 3/03/2014 7:02 \| 119 \| 49 \| 1.9 \| 1.2 \| \| 2014 \| Adult \| 112941 \| 19 \| 4/03/2014 11:19 \| 7/03/2014 7:03 \| 205 \| 52 \| 2.8 \| 1.2 \| \| 2014 \| Adult \| 112941 \| 20 \| 11/03/2014 10:24 \| 14/03/2014 7:34 \| 239 \| 90 \| 2.9 \| 4.1 \| \| 2014 \| Adult \| 112942 \| 21 \| 26/02/2014 8:01 \| 28/02/2014 1:08 \| 67 \| 24 \| 1.7 \|  \| \| 2014 \| Adult \| 112942 \| 22 \| 2/03/2014 9:25 \| 5/03/2014 3:28 \| 150 \| 36 \| 2.8 \| 2.3 \| \| 2014 \| Adult \| 112942 \| 23 \| 8/03/2014 10:37 \| 11/03/2014 2:24 \| 191 \| 57 \| 2.6 \| 3.3 \| \| 2014 \| Adult \| 112942 \| 24 \| 11/03/2014 9:36 \| 12/03/2014 3:10 \| 53 \| 23 \| 0.7 \| 0.3 \| \| 2014 \| Adult \| 112942 \| 25 \| 15/03/2014 9:14 \| 16/03/2014 0:54 \| 45 \| 22 \| 0.6 \| 3.3 \| \| 2014 \| Adult \| 112942 \| 26 \| 19/03/2014 11:47 \| 20/03/2014 23:54 \| 49 \| 19 \| 1.5 \| 3.5 \| \| 2014 \| Adult \| 112942 \| 27 \| 22/03/2014 14:20 \| 23/03/2014 0:56 \| 34 \| 19 \| 0.4 \| 1.6 \| \| 2014 \| Adult \| 112942 \| 28 \| 23/03/2014 11:36 \| 24/03/2014 23:08 \| 97 \| 38 \| 1.5 \| 0.4 \| \| 2014 \| Adult \| 112942 \| 29 \| 26/03/2014 12:05 \| 28/03/2014 1:28 \| 109 \| 39 \| 1.5 \| 1.5 \| \| 2014 \| Adult \| 112942 \| 30 \| 29/03/2014 11:16 \| 31/03/2014 0:29 \| 95 \| 28 \| 1.5 \| 1.4 \| \| 2014 \| Adult \| 112943 \| 31 \| 23/02/2014 3:37 \| 26/02/2014 4:14 \| 243 \| 97 \| 3 \|  \| \| 2014 \| Adult \| 112943 \| 32 \| 28/02/2014 8:17 \| 3/03/2014 1:46 \| 242 \| 74 \| 2.7 \| 2.2 \| \| 2014 \| Adult \| 112943 \| 33 \| 5/03/2014 7:45 \| 8/03/2014 12:16 \| 275 \| 75 \| 3.2 \| 2.2 \| \| 2014 \| Adult \| 112943 \| 34 \| 10/03/2014 11:38 \| 13/03/2014 10:11 \| 256 \| 69 \| 2.9 \| 2.0 \| \| 2014 \| Adult \| 112945 \| 35 \| 23/02/2014 18:36 \| 26/02/2014 14:07 \| 275 \| 87 \| 2.8 \|  \| \| 2014 \| Adult \| 112945 \| 36 \| 1/03/2014 15:47 \| 4/03/2014 8:17 \| 274 \| 87 \| 2.7 \| 3.1 \| \| 2014 \| Adult \| 112945 \| 37 \| 7/03/2014 14:07 \| 11/03/2014 1:26 \| 304 \| 88 \| 3.5 \| 3.2 \| \| 2014 \| Adult \| 112944 \| 38 \| 21/02/2014 6:09 \| 23/02/2014 23:57 \| 206 \| 69 \| 2.7 \|  \| \| 2014 \| Adult \| 112944 \| 39 \| 26/02/2014 4:04 \| 2/03/2014 15:36 \| 371 \| 90 \| 4.5 \| 2.2 \| \| 2014 \| Adult \| 148754 \| 40 \| 13/06/2015 13:57 \| 19/06/2015 7:20 \| 276 \| 95 \| 5.7 \|  \| \| 2015 \| Adult \| 148754 \| 41 \| 21/06/2015 11:08 \| 26/06/2015 12:39 \| 330 \| 101 \| 5 \| 2.2 \| \| 2015 \| Adult \| 148755 \| 42 \| 13/06/2015 13:17 \| 19/06/2015 4:18 \| 290 \| 103 \| 5.6 \|  \| \| 2015 \| Adult \| 148755 \| 43 \| 22/06/2015 8:00 \| 28/06/2015 4:14 \| 317 \| 106 \| 5.8 \| 3.2 \| \| 2015 \| Adult \| 148755 \| 44 \| 1/07/2015 4:40 \| 7/07/2015 3:49 \| 367 \| 103 \| 6 \| 3.0 \| \| 2015 \| Adult \| 148755 \| 45 \| 10/07/2015 8:10 \| 15/07/2015 8:29 \| 338 \| 101 \| 5 \| 3.2 \| \| 2015 \| Adult \| 148755 \| 46 \| 18/07/2015 0:56 \| 23/07/2015 4:36 \| 261 \| 104 \| 5.1 \| 2.7 \| \| 2015 \| Adult \| 148755 \| 47 \| 27/07/2015 7:27 \| 3/08/2015 12:37 \| 398 \| 136 \| 7.2 \| 4.1 \| \| 2015 \| Adult \| 148755 \| 48 \| 8/08/2015 0:54 \| 14/08/2015 4:56 \| 363 \| 101 \| 6.2 \| 4.5 \| \| 2015 \| Adult \| 148755 \| 49 \| 18/08/2015 0:55 \| 23/08/2015 15:21 \| 327 \| 104 \| 5.6 \| 3.8 \| \| 2015 \| Adult \| 148755 \| 50 \| 28/08/2015 3:54 \| 3/09/2015 12:37 \| 452 \| 147 \| 6.3 \| 4.5 \| \| 2015 \| Adult \| 148755 \| 51 \| 6/09/2015 10:29 \| 12/09/2015 14:45 \| 352 \| 127 \| 6.2 \| 2.9 \| \| 2015 \| Adult \| 148755 \| 52 \| 16/09/2015 2:19 \| 21/09/2015 8:24 \| 312 \| 105 \| 5.3 \| 3.5 \| \| 2015 \| Adult \| 148755 \| 53 \| 24/09/2015 10:03 \| 30/09/2015 13:36 \| 296 \| 106 \| 6.1 \| 3.1 \| \| 2015 \| Adult \| 148755 \| 54 \| 3/10/2015 4:26 \| 9/10/2015 12:18 \| 340 \| 109 \| 6.3 \| 2.6 \| \| 2015 \| Adult \| 148755 \| 55 \| 12/10/2015 4:38 \| 18/10/2015 7:09 \| 294 \| 101 \| 6.1 \| 2.7 \| \| 2015 \| Adult \| 148755 \| 56 \| 20/10/2015 4:32 \| 27/10/2015 11:59 \| 372 \| 95 \| 7.3 \| 1.9 \| \| 2015 \| Adult \| 148756 \| 57 \| 14/06/2015 10:39 \| 24/06/2015 7:58 \| 421 \| 113 \| 9.9 \|  \| \| 2015 \| Adult \| 148756 \| 58 \| 27/06/2015 9:01 \| 6/07/2015 7:25 \| 295 \| 124 \| 8.9 \| 3.0 \| \| 2015 \| Adult \| 148756 \| 59 \| 8/07/2015 7:03 \| 14/07/2015 15:16 \| 283 \| 107 \| 6.3 \| 2.0 \| \| 2015 \| Adult \| 148756 \| 60 \| 17/07/2015 3:45 \| 24/07/2015 13:53 \| 847 \| 800 \| 7.4 \| 2.5 \| \| 2015 \| Adult \| 148756 \| 61 \| 18/08/2015 12:19 \| 21/08/2015 4:36 \| 204 \| 150 \| 2.7 \| 24.9 \| \| 2015 \| Adult \| 148756 \| 62 \| 22/08/2015 7:39 \| 2/09/2015 2:42 \| 878 \| 759 \| 10.8 \| 1.1 \| \| 2015 \| Adult \| 148756 \| 63 \| 4/09/2015 12:42 \| 11/09/2015 12:36 \| 467 \| 157 \| 7 \| 2.4 \| \| 2015 \| Adult \| 148759 \| 64 \| 11/06/2015 14:14 \| 22/06/2015 10:43 \| 386 \| 136 \| 10.8 \|  \| \| 2015 \| Adult \| 148759 \| 65 \| 26/06/2015 11:29 \| 3/07/2015 16:33 \| 432 \| 135 \| 7.2 \| 4.0 \| \| 2015 \| Adult \| 148759 \| 66 \| 8/07/2015 6:18 \| 15/07/2015 10:25 \| 475 \| 135 \| 7.2 \| 4.6 \| \| 2015 \| Adult \| 148759 \| 67 \| 20/07/2015 0:53 \| 26/07/2015 17:39 \| 413 \| 136 \| 6.7 \| 4.6 \| \| 2015 \| Adult \| 148759 \| 68 \| 31/07/2015 4:01 \| 8/08/2015 18:23 \| 555 \| 142 \| 8.6 \| 4.4 \| \| 2015 \| Adult \| 148759 \| 69 \| 14/08/2015 4:37 \| 22/08/2015 19:10 \| 534 \| 149 \| 8.6 \| 5.4 \| \| 2015 \| Adult \| 148759 \| 70 \| 27/08/2015 7:35 \| 4/09/2015 15:20 \| 506 \| 139 \| 8.3 \| 4.5 \| \| 2015 \| Adult \| 148759 \| 71 \| 11/09/2015 0:11 \| 20/09/2015 20:16 \| 492 \| 143 \| 9.8 \| 6.4 \| \| 2011 \| Juvenile \| 1543 \| 72 \| 15/05/2011 4:23 \| 18/05/2011 5:18 \| 134 \| 52 \| 3 \|  \| \| 2011 \| Juvenile \| 1543 \| 73 \| 20/05/2011 9:40 \| 24/05/2011 6:08 \| 245 \| 80 \| 3.8 \| 2.2 \| \| 2011 \| Juvenile \| 1543 \| 74 \| 27/05/2011 9:21 \| 30/05/2011 21:07 \| 246 \| 65 \| 3.5 \| 3.1 \| \| 2011 \| Juvenile \| 1543 \| 75 \| 2/06/2011 4:21 \| 5/06/2011 10:03 \| 169 \| 54 \| 3.2 \| 2.3 \| \| 2011 \| Juvenile \| 1543 \| 76 \| 6/06/2011 13:03 \| 9/06/2011 3:24 \| 190 \| 60 \| 2.6 \| 1.1 \| \| 2011 \| Juvenile \| 1543 \| 77 \| 10/06/2011 11:22 \| 13/06/2011 5:54 \| 150 \| 62 \| 2.8 \| 1.3 \| \| 2011 \| Juvenile \| 1543 \| 78 \| 15/06/2011 14:25 \| 20/06/2011 9:11 \| 290 \| 127 \| 4.8 \| 2.4 \| \| 2011 \| Juvenile \| 2162 \| 79 \| 17/05/2011 7:29 \| 20/05/2011 0:14 \| 180 \| 66 \| 2.7 \|  \| \| 2011 \| Juvenile \| 2162 \| 80 \| 21/05/2011 11:46 \| 25/05/2011 5:12 \| 176 \| 62 \| 3.7 \| 1.5 \| \| 2011 \| Juvenile \| 2162 \| 81 \| 27/05/2011 12:44 \| 30/05/2011 6:32 \| 130 \| 58 \| 2.7 \| 2.3 \| \| 2011 \| Juvenile \| 2162 \| 82 \| 1/06/2011 20:20 \| 4/06/2011 16:19 \| 153 \| 59 \| 2.8 \| 2.6 \| \| 2011 \| Juvenile \| 2162 \| 83 \| 7/06/2011 13:53 \| 11/06/2011 2:42 \| 215 \| 65 \| 3.5 \| 2.9 \| \| 2011 \| Juvenile \| 2162 \| 84 \| 13/06/2011 19:46 \| 18/06/2011 6:39 \| 214 \| 74 \| 4.4 \| 2.7 \| \| 2011 \| Juvenile \| 2162 \| 85 \| 18/06/2011 8:25 \| 22/06/2011 2:33 \| 138 \| 53 \| 3.8 \| 0.1 \| \| 2011 \| Juvenile \| 2162 \| 86 \| 25/06/2011 9:51 \| 28/06/2011 6:33 \| 227 \| 92 \| 2.8 \| 3.3 \| \| 2011 \| Juvenile \| 2162 \| 87 \| 30/06/2011 6:09 \| 2/07/2011 5:42 \| 171 \| 75 \| 2 \| 2.0 \| \| 2011 \| Juvenile \| 2162 \| 88 \| 4/07/2011 11:12 \| 6/07/2011 7:23 \| 128 \| 58 \| 1.8 \| 2.2 \| \| 2011 \| Juvenile \| 2162 \| 89 \| 9/07/2011 2:55 \| 10/07/2011 11:22 \| 179 \| 68 \| 1.3 \| 2.8 \| \| 2011 \| Juvenile \| 2162 \| 90 \| 10/07/2011 12:29 \| 12/07/2011 2:28 \| 93 \| 39 \| 1.3 \| 0.0 \| \| 2011 \| Juvenile \| 2162 \| 91 \| 15/07/2011 4:17 \| 18/07/2011 7:36 \| 139 \| 51 \| 3.1 \| 3.1 \| \| 2011 \| Juvenile \| 2162 \| 92 \| 21/07/2011 19:51 \| 24/07/2011 6:49 \| 121 \| 52 \| 2.4 \| 3.5 \| \| 2011 \| Juvenile \| 2162 \| 93 \| 26/07/2011 1:45 \| 30/07/2011 8:56 \| 169 \| 54 \| 4.3 \| 1.8 \| \| 2011 \| Juvenile \| 2162 \| 94 \| 2/08/2011 5:29 \| 3/08/2011 6:49 \| 122 \| 54 \| 1 \| 2.9 \| \| 2011 \| Juvenile \| 2162 \| 95 \| 5/08/2011 7:02 \| 6/08/2011 20:28 \| 158 \| 89 \| 2 \| 2.0 \| \| 2011 \| Juvenile \| 2162 \| 96 \| 7/08/2011 7:56 \| 9/08/2011 2:22 \| 145 \| 55 \| 1.8 \| 0.5 \| \| 2011 \| Juvenile \| 2162 \| 97 \| 10/08/2011 5:05 \| 12/08/2011 7:35 \| 162 \| 56 \| 2.1 \| 1.1 \| \| 2011 \| Juvenile \| 6074 \| 98 \| 18/05/2011 8:45 \| 24/05/2011 3:17 \| 338 \| 90 \| 5.8 \|  \| \| 2011 \| Juvenile \| 6074 \| 99 \| 27/05/2011 10:36 \| 31/05/2011 4:43 \| 318 \| 100 \| 3.8 \| 3.3 \| \| 2011 \| Juvenile \| 6074 \| 100 \| 2/06/2011 1:25 \| 4/06/2011 17:58 \| 191 \| 66 \| 2.7 \| 1.9 \| \| 2011 \| Juvenile \| 6074 \| 101 \| 6/06/2011 9:44 \| 11/06/2011 10:58 \| 367 \| 98 \| 5 \| 1.7 \| \| 2011 \| Juvenile \| 6074 \| 102 \| 15/06/2011 21:22 \| 19/06/2011 7:35 \| 231 \| 111 \| 3.4 \| 4.4 \| \| 2011 \| Juvenile \| 6074 \| 103 \| 23/06/2011 3:16 \| 27/06/2011 10:18 \| 328 \| 147 \| 4.3 \| 3.8 \| \| 2011 \| Juvenile \| 6074 \| 104 \| 29/06/2011 10:40 \| 1/07/2011 21:37 \| 214 \| 118 \| 2.4 \| 2.0 \| \| 2011 \| Juvenile \| 6074 \| 105 \| 4/07/2011 16:02 \| 9/07/2011 16:49 \| 645 \| 346 \| 5 \| 2.8 \| \| 2011 \| Juvenile \| 6074 \| 106 \| 15/07/2011 1:05 \| 19/07/2011 1:14 \| 308 \| 173 \| 4 \| 5.3 \| \| 2011 \| Juvenile \| 6074 \| 107 \| 20/07/2011 22:58 \| 27/07/2011 7:19 \| 266 \| 82 \| 6.3 \| 1.9 \| \| 2011 \| Juvenile \| 6074 \| 108 \| 29/07/2011 22:00 \| 2/08/2011 5:20 \| 253 \| 76 \| 3.3 \| 2.6 \| \| 2011 \| Juvenile \| 6074 \| 109 \| 5/08/2011 3:40 \| 9/08/2011 2:27 \| 193 \| 68 \| 3.9 \| 2.9 \| \| 2011 \| Juvenile \| 6074 \| 110 \| 10/08/2011 9:05 \| 11/08/2011 3:55 \| 102 \| 50 \| 0.8 \| 1.3 \| \| 2011 \| Juvenile \| 6074 \| 111 \| 13/08/2011 11:22 \| 18/08/2011 14:02 \| 307 \| 88 \| 5.1 \| 2.3 \| \| 2011 \| Juvenile \| 6074 \| 112 \| 23/08/2011 17:27 \| 27/08/2011 2:31 \| 308 \| 100 \| 3.4 \| 5.1 \| \| 2011 \| Juvenile \| 6074 \| 113 \| 30/08/2011 9:47 \| 7/09/2011 19:31 \| 712 \| 200 \| 8.4 \| 3.3 \| \| 2011 \| Juvenile \| 6074 \| 114 \| 10/09/2011 10:26 \| 18/09/2011 16:15 \| 711 \| 136 \| 8.2 \| 2.6 \| \| 2011 \| Juvenile \| 6074 \| 115 \| 24/09/2011 18:10 \| 2/10/2011 11:49 \| 680 \| 232 \| 7.7 \| 6.1 \| \| 2011 \| Juvenile \| 6074 \| 116 \| 11/10/2011 5:01 \| 18/10/2011 17:10 \| 610 \| 266 \| 7.5 \| 8.7 \| \| 2011 \| Juvenile \| 103751 \| 117 \| 16/05/2011 1:46 \| 22/05/2011 13:52 \| 529 \| 214 \| 6.5 \|  \| \| 2011 \| Juvenile \| 103751 \| 118 \| 26/05/2011 12:54 \| 2/06/2011 4:30 \| 681 \| 209 \| 6.6 \| 4.0 \| \| 2011 \| Juvenile \| 103751 \| 119 \| 5/06/2011 0:15 \| 10/06/2011 16:55 \| 275 \| 98 \| 5.7 \| 2.8 \| \| 2011 \| Juvenile \| 103751 \| 120 \| 13/06/2011 13:38 \| 19/06/2011 5:53 \| 349 \| 108 \| 5.7 \| 2.9 \| \| 2011 \| Juvenile \| 103751 \| 121 \| 23/06/2011 15:01 \| 29/06/2011 19:04 \| 500 \| 135 \| 6.2 \| 4.4 \| \| 2011 \| Juvenile \| 68025 \| 122 \| 3/06/2012 5:23 \| 9/06/2012 19:48 \| 269 \| 90 \| 6.6 \|  \| \| 2011 \| Juvenile \| 68025 \| 123 \| 14/06/2012 2:12 \| 18/06/2012 1:33 \| 260 \| 131 \| 4 \| 4.3 \| \| 2011 \| Juvenile \| 68025 \| 124 \| 19/06/2012 23:04 \| 24/06/2012 2:02 \| 265 \| 91 \| 4.1 \| 1.9 \| \| 2011 \| Juvenile \| 68025 \| 125 \| 26/06/2012 3:01 \| 29/06/2012 5:50 \| 215 \| 85 \| 3.1 \| 2.0 \| \| 2011 \| Juvenile \| 68025 \| 126 \| 1/07/2012 23:24 \| 7/07/2012 5:45 \| 253 \| 77 \| 5.3 \| 2.7 \| \| 2011 \| Juvenile \| 68025 \| 127 \| 9/07/2012 5:40 \| 11/07/2012 2:31 \| 98 \| 41 \| 1.8 \| 2.0 \| \| 2011 \| Juvenile \| 68025 \| 128 \| 12/07/2012 2:29 \| 14/07/2012 1:55 \| 108 \| 51 \| 2 \| 1.0 \| \| 2011 \| Juvenile \| 68025 \| 129 \| 16/07/2012 1:31 \| 19/07/2012 5:36 \| 141 \| 58 \| 3.2 \| 2.0 \| \| 2011 \| Juvenile \| 68025 \| 130 \| 21/07/2012 22:34 \| 25/07/2012 5:51 \| 254 \| 94 \| 3.3 \| 2.7 \| |  |  |  |  |  |  |  |  |  |
| --- | --- | --- | --- | --- | --- | --- | --- | --- | --- | --- | --- | --- | --- | --- | --- | --- | --- | --- | --- | --- | --- | --- | --- | --- | --- | --- | --- | --- | --- | --- | --- | --- | --- | --- | --- | --- | --- | --- | --- | --- | --- | --- | --- | --- | --- | --- | --- | --- | --- | --- | --- | --- | --- | --- | --- | --- | --- | --- | --- | --- | --- | --- | --- | --- | --- | --- | --- | --- | --- | --- | --- | --- | --- | --- | --- | --- | --- | --- | --- | --- | --- | --- | --- | --- | --- | --- | --- | --- | --- | --- | --- | --- | --- | --- | --- | --- | --- | --- | --- | --- | --- | --- | --- | --- | --- | --- | --- | --- | --- | --- | --- | --- | --- | --- | --- | --- | --- | --- | --- | --- | --- | --- | --- | --- | --- | --- | --- | --- | --- | --- | --- | --- | --- | --- | --- | --- | --- | --- | --- | --- | --- | --- | --- | --- | --- | --- | --- | --- | --- | --- | --- | --- | --- | --- | --- | --- | --- | --- | --- | --- | --- | --- | --- | --- | --- | --- | --- | --- | --- | --- | --- | --- | --- | --- | --- | --- | --- | --- | --- | --- | --- | --- | --- | --- | --- | --- | --- | --- | --- | --- | --- | --- | --- | --- | --- | --- | --- | --- | --- | --- | --- | --- | --- | --- | --- | --- | --- | --- | --- | --- | --- | --- | --- | --- | --- | --- | --- | --- | --- | --- | --- | --- | --- | --- | --- | --- | --- | --- | --- | --- | --- | --- | --- | --- | --- | --- | --- | --- | --- | --- | --- | --- | --- | --- | --- | --- | --- | --- | --- | --- | --- | --- | --- | --- | --- | --- | --- | --- | --- | --- | --- | --- | --- | --- | --- | --- | --- | --- | --- | --- | --- | --- | --- | --- | --- | --- | --- | --- | --- | --- | --- | --- | --- | --- | --- | --- | --- | --- | --- | --- | --- | --- | --- | --- | --- | --- | --- | --- | --- | --- | --- | --- | --- | --- | --- | --- | --- | --- | --- | --- | --- | --- | --- | --- | --- | --- | --- | --- | --- | --- | --- | --- | --- | --- | --- | --- | --- | --- | --- | --- | --- | --- | --- | --- | --- | --- | --- | --- | --- | --- | --- | --- | --- | --- | --- | --- | --- | --- | --- | --- | --- | --- | --- | --- | --- | --- | --- | --- | --- | --- | --- | --- | --- | --- | --- | --- | --- | --- | --- | --- | --- | --- | --- | --- | --- | --- | --- | --- | --- | --- | --- | --- | --- | --- | --- | --- | --- | --- | --- | --- | --- | --- | --- | --- | --- | --- | --- | --- | --- | --- | --- | --- | --- | --- | --- | --- | --- | --- | --- | --- | --- | --- | --- | --- | --- | --- | --- | --- | --- | --- | --- | --- | --- | --- | --- | --- | --- | --- | --- | --- | --- | --- | --- | --- | --- | --- | --- | --- | --- | --- | --- | --- | --- | --- | --- | --- | --- | --- | --- | --- | --- | --- | --- | --- | --- | --- | --- | --- | --- | --- | --- | --- | --- | --- | --- | --- | --- | --- | --- | --- | --- | --- | --- | --- | --- | --- | --- | --- | --- | --- | --- | --- | --- | --- | --- | --- | --- | --- | --- | --- | --- | --- | --- | --- | --- | --- | --- | --- | --- | --- | --- | --- | --- | --- | --- | --- | --- | --- | --- | --- | --- | --- | --- | --- | --- | --- | --- | --- | --- | --- | --- | --- | --- | --- | --- | --- | --- | --- | --- | --- | --- | --- | --- | --- | --- | --- | --- | --- | --- | --- | --- | --- | --- | --- | --- | --- | --- | --- | --- | --- | --- | --- | --- | --- | --- | --- | --- | --- | --- | --- | --- | --- | --- | --- | --- | --- | --- | --- | --- | --- | --- | --- | --- | --- | --- | --- | --- | --- | --- | --- | --- | --- | --- | --- | --- | --- | --- | --- | --- | --- | --- | --- | --- | --- | --- | --- | --- | --- | --- | --- | --- | --- | --- | --- | --- | --- | --- | --- | --- | --- | --- | --- | --- | --- | --- | --- | --- | --- | --- | --- | --- | --- | --- | --- | --- | --- | --- | --- | --- | --- | --- | --- | --- | --- | --- | --- | --- | --- | --- | --- | --- | --- | --- | --- | --- | --- | --- | --- | --- | --- | --- | --- | --- | --- | --- | --- | --- | --- | --- | --- | --- | --- | --- | --- | --- | --- | --- | --- | --- | --- | --- | --- | --- | --- | --- | --- | --- | --- | --- | --- | --- | --- | --- | --- | --- | --- | --- | --- | --- | --- | --- | --- | --- | --- | --- | --- | --- | --- | --- | --- | --- | --- | --- | --- | --- | --- | --- | --- | --- | --- | --- | --- | --- | --- | --- | --- | --- | --- | --- | --- | --- | --- | --- | --- | --- | --- | --- | --- | --- | --- | --- | --- | --- | --- | --- | --- | --- | --- | --- | --- | --- | --- | --- | --- | --- | --- | --- | --- | --- | --- | --- | --- | --- | --- | --- | --- | --- | --- | --- | --- | --- | --- | --- | --- | --- | --- | --- | --- | --- | --- | --- | --- | --- | --- | --- | --- | --- | --- | --- | --- | --- | --- | --- | --- | --- | --- | --- | --- | --- | --- | --- | --- | --- | --- | --- | --- | --- | --- | --- | --- | --- | --- | --- | --- | --- | --- | --- | --- | --- | --- | --- | --- | --- | --- | --- | --- | --- | --- | --- | --- | --- | --- | --- | --- | --- | --- | --- | --- | --- | --- | --- | --- | --- | --- | --- | --- | --- | --- | --- | --- | --- | --- | --- | --- | --- | --- | --- | --- | --- | --- | --- | --- | --- | --- | --- | --- | --- | --- | --- | --- | --- | --- | --- | --- | --- | --- | --- | --- | --- | --- | --- | --- | --- | --- | --- | --- | --- | --- | --- | --- | --- | --- | --- | --- | --- | --- | --- | --- | --- | --- | --- | --- | --- | --- | --- | --- | --- | --- | --- | --- | --- | --- | --- | --- | --- | --- | --- | --- | --- | --- | --- | --- | --- | --- | --- | --- | --- | --- | --- | --- | --- | --- | --- | --- | --- | --- | --- | --- | --- | --- | --- | --- | --- | --- | --- | --- | --- | --- | --- | --- | --- | --- | --- | --- | --- | --- | --- | --- | --- | --- | --- | --- | --- | --- | --- | --- | --- | --- | --- | --- | --- | --- | --- | --- | --- | --- | --- | --- | --- | --- | --- | --- | --- | --- | --- | --- | --- | --- | --- | --- | --- | --- | --- | --- | --- | --- | --- | --- | --- | --- | --- | --- | --- | --- | --- | --- | --- | --- | --- | --- | --- | --- | --- | --- | --- | --- | --- | --- | --- | --- | --- | --- | --- | --- | --- | --- | --- | --- | --- | --- | --- | --- | --- | --- | --- | --- | --- | --- | --- | --- | --- | --- | --- | --- | --- | --- | --- | --- | --- | --- | --- | --- | --- | --- | --- | --- | --- | --- | --- | --- | --- | --- | --- | --- | --- | --- | --- | --- | --- | --- | --- | --- | --- | --- | --- | --- | --- | --- | --- | --- | --- | --- | --- | --- | --- | --- | --- | --- | --- | --- | --- | --- | --- | --- | --- | --- | --- | --- | --- | --- | --- | --- | --- | --- | --- | --- | --- | --- | --- | --- | --- | --- | --- | --- | --- | --- | --- | --- | --- | --- | --- | --- | --- | --- | --- | --- | --- | --- | --- | --- | --- | --- | --- | --- | --- | --- | --- | --- | --- | --- | --- | --- | --- | --- | --- | --- | --- | --- | --- | --- | --- | --- | --- | --- | --- | --- | --- | --- | --- | --- | --- | --- | --- | --- | --- | --- | --- | --- | --- | --- | --- | --- | --- | --- | --- | --- | --- | --- | --- | --- | --- | --- | --- | --- | --- | --- | --- | --- | --- | --- | --- | --- | --- | --- | --- | --- | --- | --- | --- | --- | --- | --- | --- | --- | --- | --- | --- | --- | --- | --- | --- | --- | --- | --- | --- | --- | --- | --- | --- | --- | --- | --- | --- | --- | --- | --- | --- | --- | --- | --- | --- | --- | --- | --- | --- | --- | --- | --- | --- | --- | --- | --- | --- | --- | --- | --- | --- | --- | --- | --- | --- | --- | --- | --- | --- | --- | --- | --- | --- | --- | --- | --- | --- | --- | --- | --- | --- | --- | --- | --- | --- | --- | --- | --- | --- | --- | --- | --- | --- | --- | --- | --- | --- | --- | --- | --- | --- | --- | --- | --- | --- | --- | --- | --- | --- | --- | --- | --- | --- | --- | --- | --- | --- | --- | --- | --- | --- | --- | --- | --- | --- | --- | --- | --- | --- | --- | --- | --- | --- | --- | --- | --- | --- | --- | --- | --- | --- | --- | --- | --- |

|  |  |  |  |  |  |  |  |  |  |
| --- | --- | --- | --- | --- | --- | --- | --- | --- | --- |


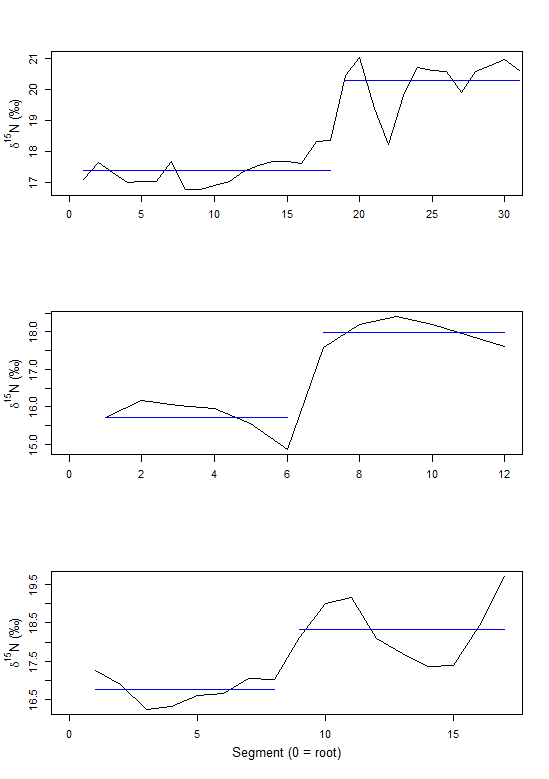


**Fig S1:** Three juvenile male South American sea lion vibrissae had changes in nitrogen (δ^15^N) values that were consistent with weaning. We used change point analysis to objectively identify a change in δ^15^N values along the whisker. The vibrissae segments closer to the root were assumed to represent nutritional independence.


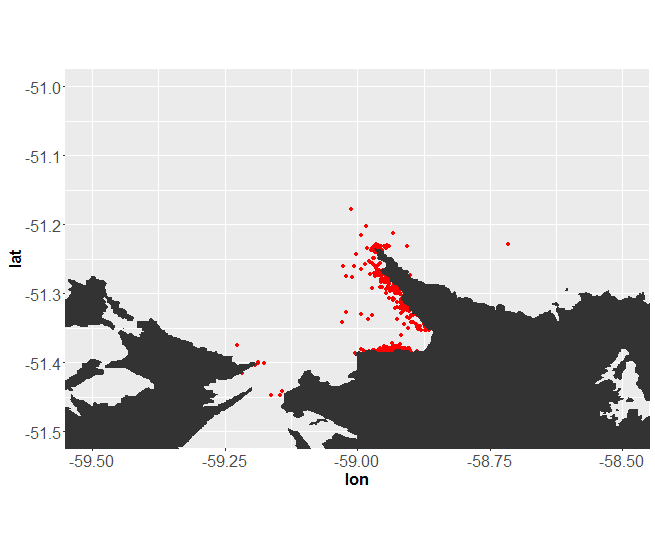


**Fig S2:** One juvenile male SASL remained within close proximity to land for the duration of the deployment period (max distance from land 13.5 km, mean 1.2 ± 1.8 km, red dots). We could not reliably define the start and end of foraging trips and therefore, this individual was excluded from movement analysis.


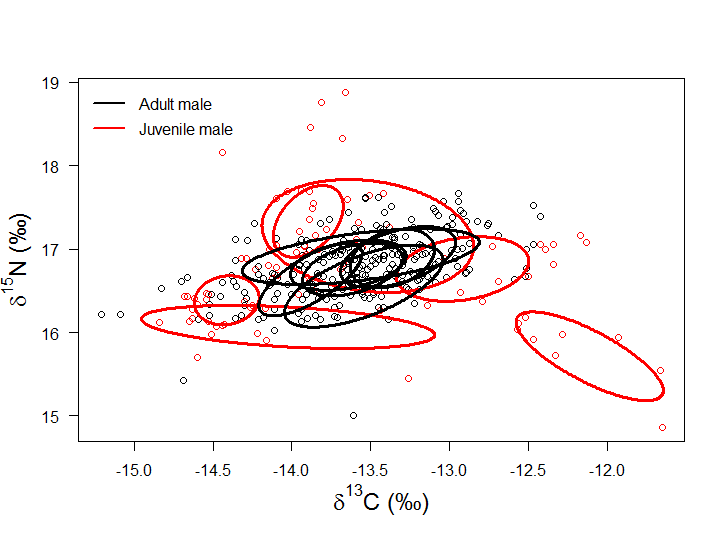


**Fig S3:** Standard Ellipse Areas for 13 South American sea lion (*Otaria flavescens*) vibrissae (Black ellipses = adult male, and Red = juvenile male). Standard Ellipse Areas are a proxy for core isotopic area (analogous to SD for univariate data).
